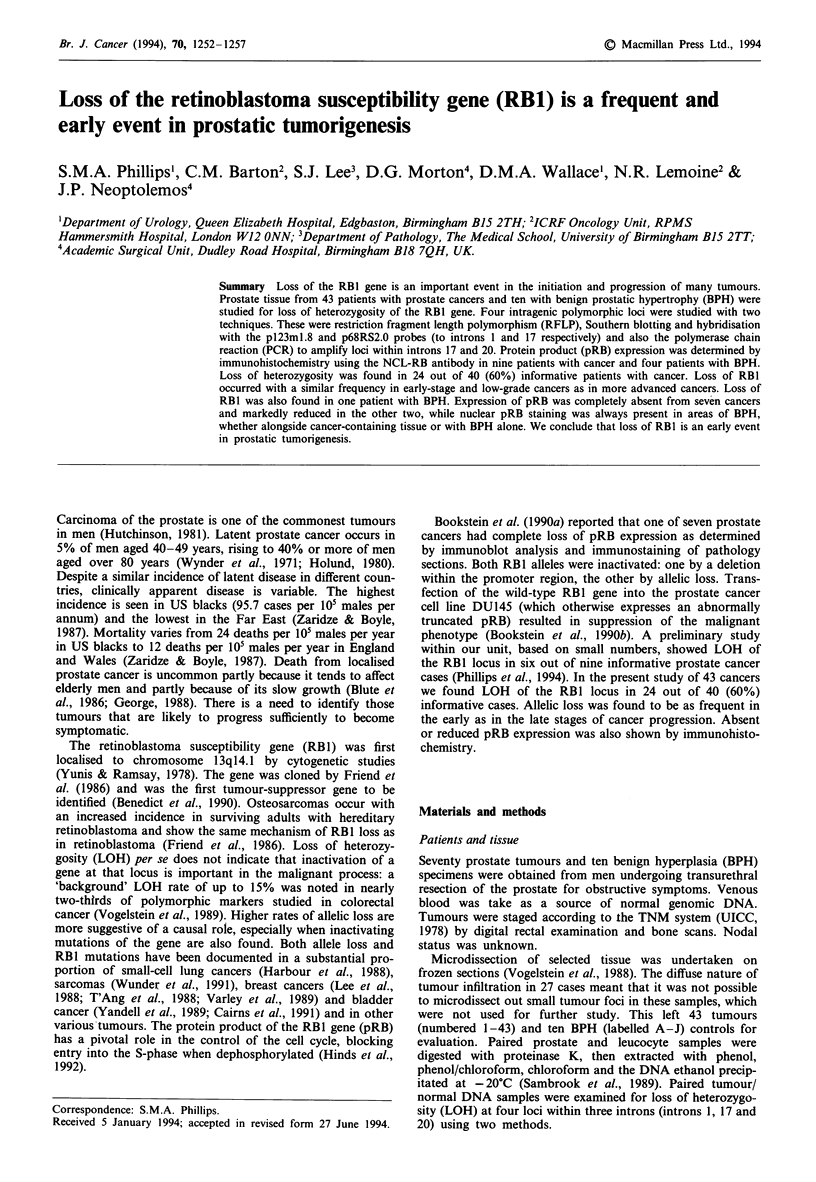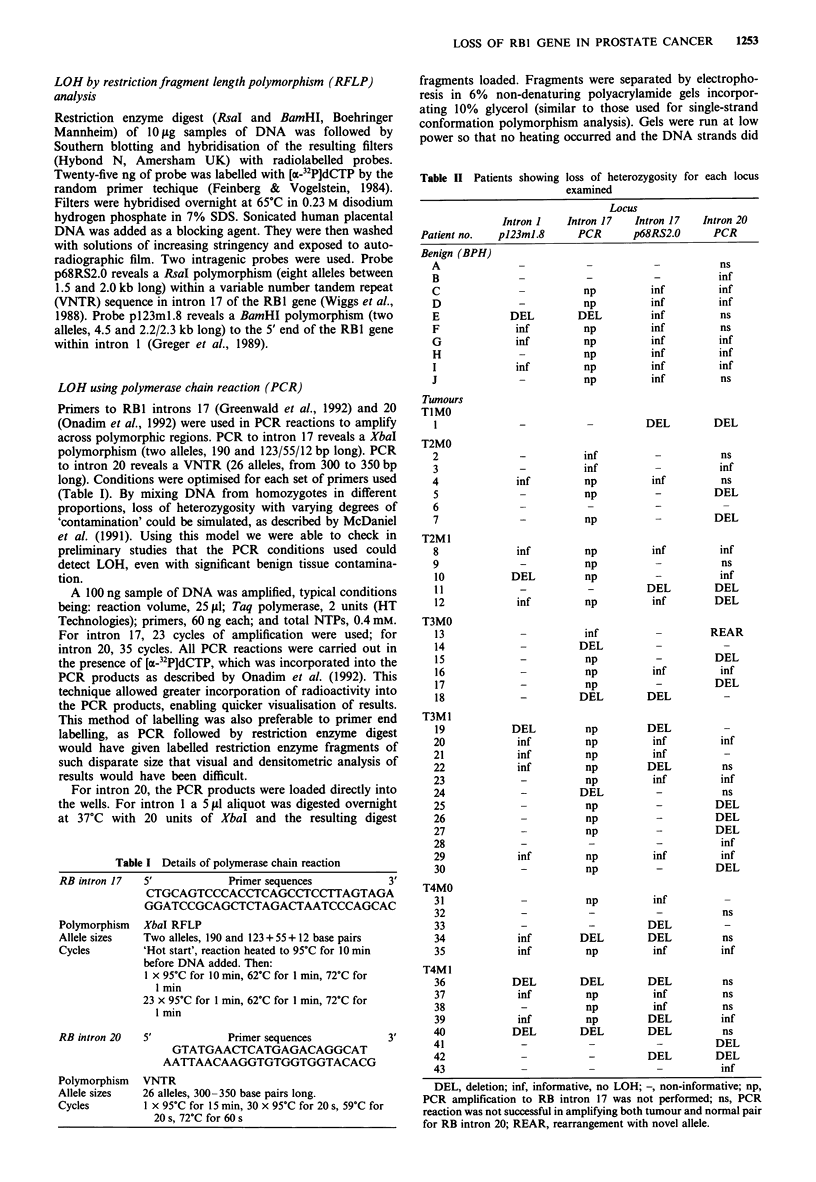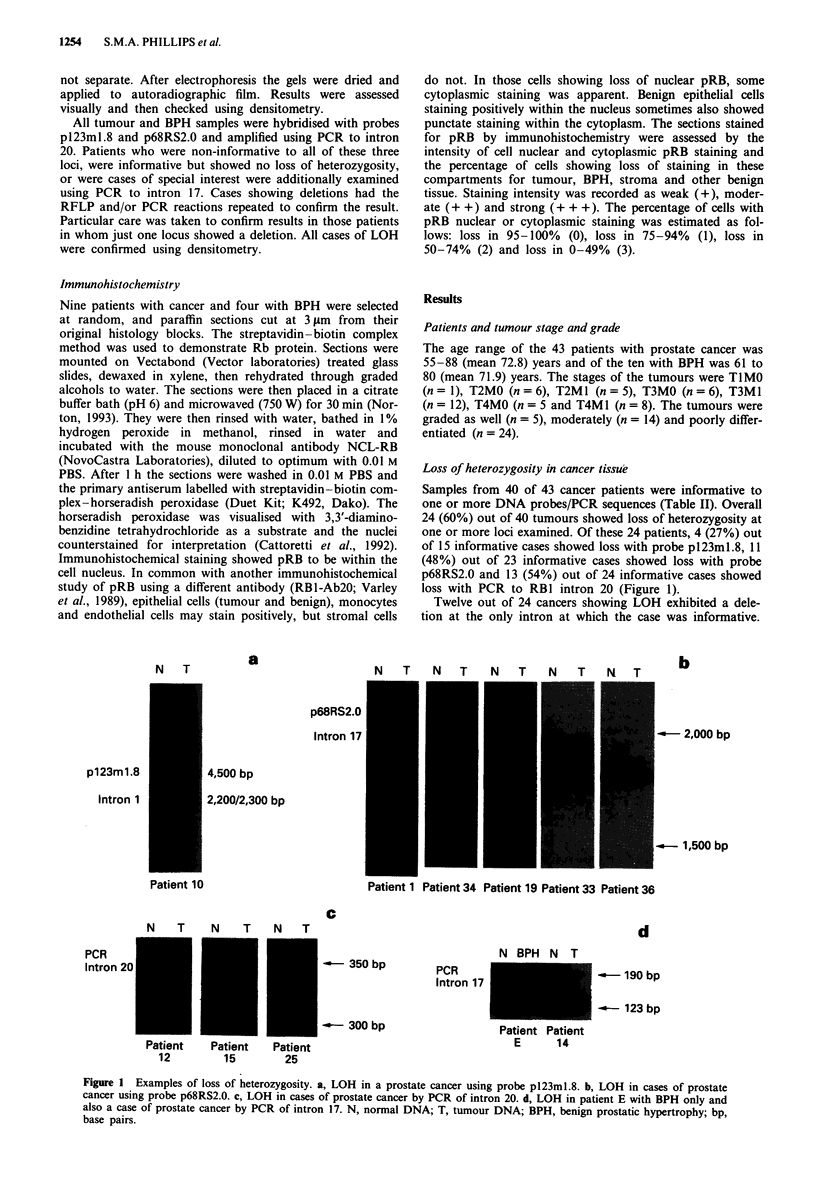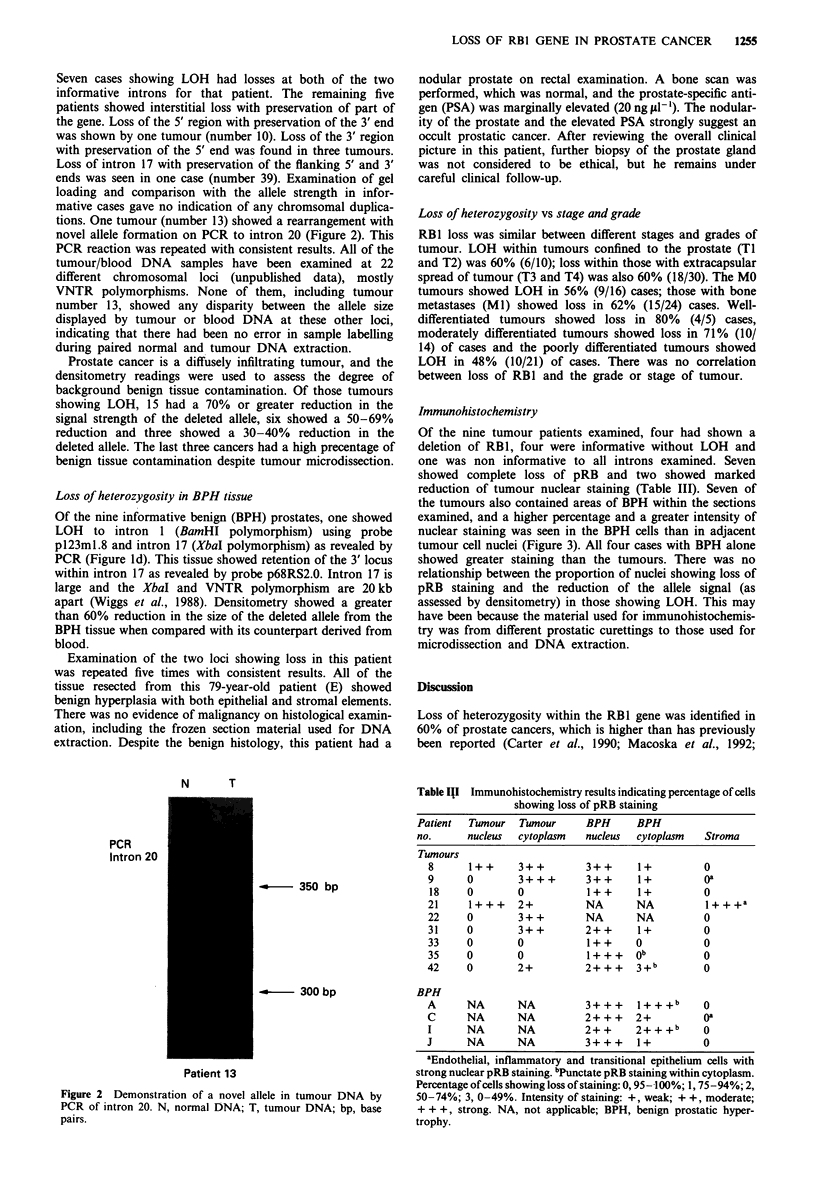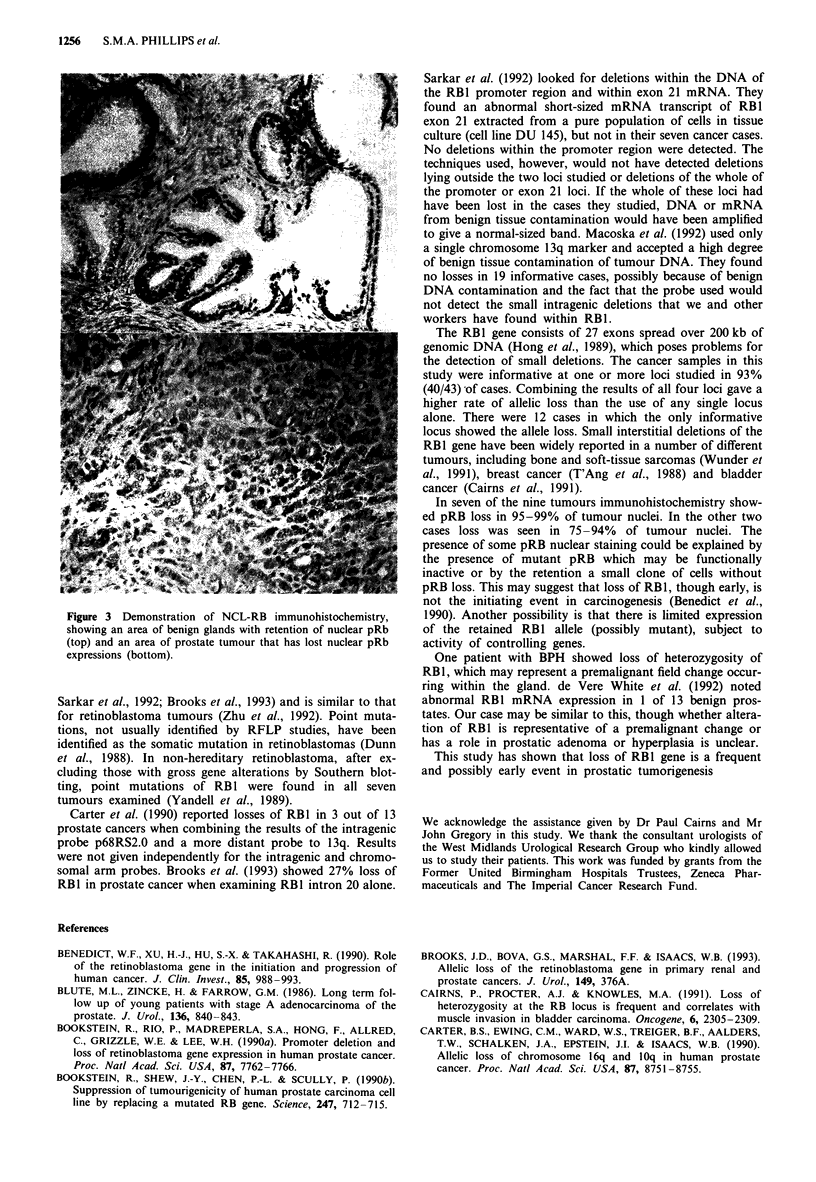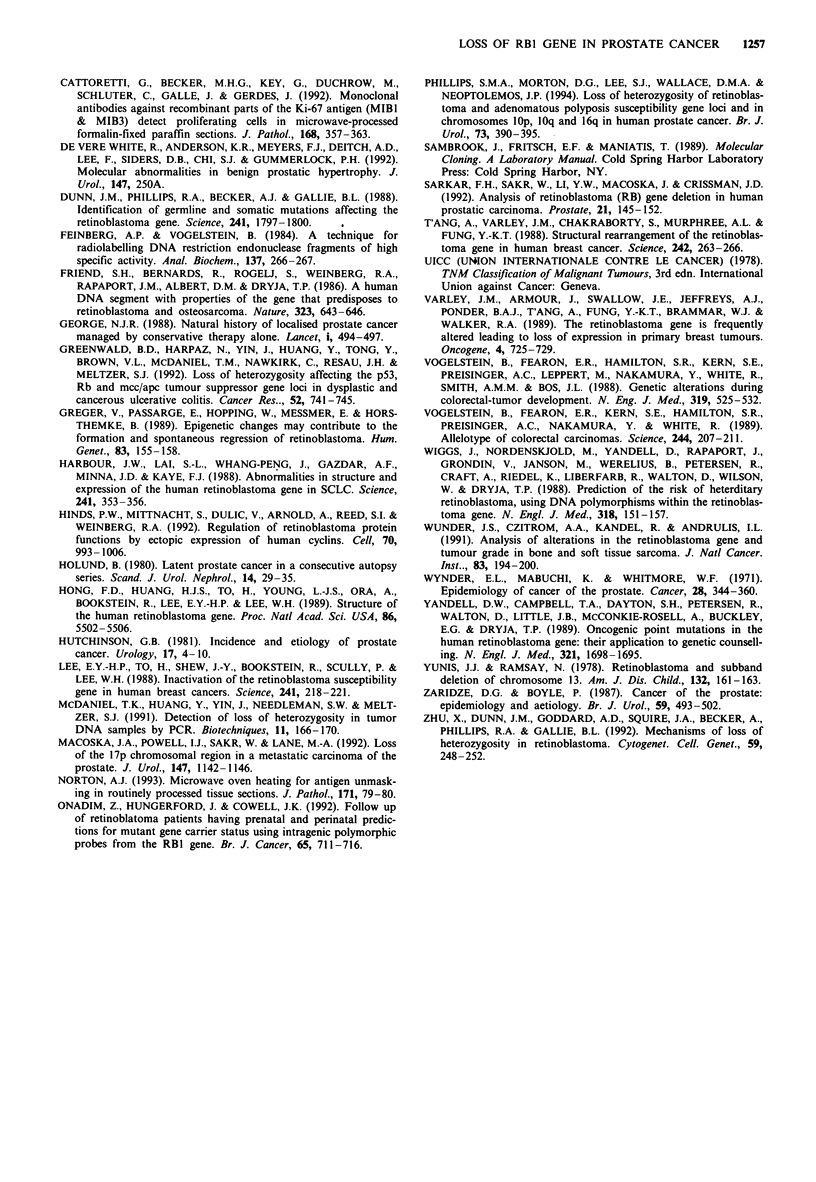# Loss of the retinoblastoma susceptibility gene (RB1) is a frequent and early event in prostatic tumorigenesis.

**DOI:** 10.1038/bjc.1994.482

**Published:** 1994-12

**Authors:** S. M. Phillips, C. M. Barton, S. J. Lee, D. G. Morton, D. M. Wallace, N. R. Lemoine, J. P. Neoptolemos

**Affiliations:** Department of Urology, Queen Elizabeth Hospital, Edgbaston, Birmingham, UK.

## Abstract

**Images:**


Br. J. Cancer (1994), 70, 1252–1257 © Macmillan Press Ltd., 1994

### Loss of the retinoblastoma susceptibility gene (RB1) is a frequent and early event in prostatic tumorigenesis

S.M.A. Phillips[1], C.M. Barton[2], S.J. Lee[3], D.G. Morton[4], D.M.A. Wallace[1], N.R. Lemoine[2] & J.P. Neoptolemos[4]

[1]*Department of Urology, Queen Elizabeth Hospital, Edgbaston, Birmingham B15 2TH;* [2]*ICRF Oncology Unit, RPMS Hammersmith Hospital, London W12 0NN;* [3]*Department of Pathology, The Medical School, University of Birmingham B15 2TT;* [4]*Academic Surgical Unit, Dudley Road Hospital, Birmingham B18 7QH, UK.*

**Summary** Loss of the RB1 gene is an important event in the initiation and progression of many tumours. Prostate tissue from 43 patients with prostate cancers and ten with benign prostatic hypertrophy (BPH) were studied for loss of heterozygosity of the RB1 gene. Four intragenic polymorphic loci were studied with two techniques. These were restriction fragment length polymorphism (RFLP), Southern blotting and hybridisation with the p123m1.8 and p68RS2.0 probes (to introns 1 and 17 respectively) and also the polymerase chain reaction (PCR) to amplify loci within introns 17 and 20. Protein product (pRB) expression was determined by immunohistochemistry using the NCL-RB antibody in nine patients with cancer and four patients with BPH. Loss of heterozygosity was found in 24 out of 40 (60%) informative patients with cancer. Loss of RB1 occurred with a similar frequency in early-stage and low-grade cancers as in more advanced cancers. Loss of RB1 was also found in one patient with BPH. Expression of pRB was completely absent from seven cancers and markedly reduced in the other two, while nuclear pRB staining was always present in areas of BPH, whether alongside cancer-containing tissue or with BPH alone. We conclude that loss of RB1 is an early event in prostatic tumorigenesis.

Carcinoma of the prostate is one of the commonest tumours in men (Hutchinson, 1981). Latent prostate cancer occurs in 5% of men aged 40–49 years, rising to 40% or more of men aged over 80 years (Wynder *et al.*, 1971; Holund, 1980). Despite a similar incidence of latent disease in different countries, clinically apparent disease is variable. The highest incidence is seen in US blacks (95.7 cases per $10^5$ males per annum) and the lowest in the Far East (Zaridze & Boyle, 1987). Mortality varies from 24 deaths per $10^5$ males per year in US blacks to 12 deaths per $10^5$ males per year in England and Wales (Zaridze & Boyle, 1987). Death from localised prostate cancer is uncommon partly because it tends to affect elderly men and partly because of its slow growth (Blute *et al.*, 1986; George, 1988). There is a need to identify those tumours that are likely to progress sufficiently to become symptomatic.

The retinoblastoma susceptibility gene (RB1) was first localised to chromosome 13q14.1 by cytogenetic studies (Yunis & Ramsay, 1978). The gene was cloned by Friend *et al.* (1986) and was the first tumour-suppressor gene to be identified (Benedict *et al.*, 1990). Osteosarcomas occur with an increased incidence in surviving adults with hereditary retinoblastoma and show the same mechanism of RB1 loss as in retinoblastoma (Friend *et al.*, 1986). Loss of heterozygosity (LOH) *per se* does not indicate that inactivation of a gene at that locus is important in the malignant process: a 'background' LOH rate of up to 15% was noted in nearly two-thirds of polymorphic markers studied in colorectal cancer (Vogelstein *et al.*, 1989). Higher rates of allelic loss are more suggestive of a causal role, especially when inactivating mutations of the gene are also found. Both allele loss and RB1 mutations have been documented in a substantial proportion of small-cell lung cancers (Harbour *et al.*, 1988), sarcomas (Wunder *et al.*, 1991), breast cancers (Lee *et al.*, 1988; T'Ang *et al.*, 1988; Varley *et al.*, 1989) and bladder cancer (Yandell *et al.*, 1989; Cairns *et al.*, 1991) and in other various tumours. The protein product of the RB1 gene (pRB) has a pivotal role in the control of the cell cycle, blocking entry into the S-phase when dephosphorylated (Hinds *et al.*, 1992).

Correspondence: S.M.A. Phillips.
Received 5 January 1994; accepted in revised form 27 June 1994.

Bookstein *et al.* (1990*a*) reported that one of seven prostate cancers had complete loss of pRB expression as determined by immunoblot analysis and immunostaining of pathology sections. Both RB1 alleles were inactivated: one by a deletion within the promoter region, the other by allelic loss. Transfection of the wild-type RB1 gene into the prostate cancer cell line DU145 (which otherwise expresses an abnormally truncated pRB) resulted in suppression of the malignant phenotype (Bookstein *et al.*, 1990*b*). A preliminary study within our unit, based on small numbers, showed LOH of the RB1 locus in six out of nine informative prostate cancer cases (Phillips *et al.*, 1994). In the present study of 43 cancers we found LOH of the RB1 locus in 24 out of 40 (60%) informative cases. Allelic loss was found to be as frequent in the early as in the late stages of cancer progression. Absent or reduced pRB expression was also shown by immunohistochemistry.

### Materials and methods

### *Patients and tissue*

Seventy prostate tumours and ten benign hyperplasia (BPH) specimens were obtained from men undergoing transurethral resection of the prostate for obstructive symptoms. Venous blood was take as a source of normal genomic DNA. Tumours were staged according to the TNM system (UICC, 1978) by digital rectal examination and bone scans. Nodal status was unknown.

Microdissection of selected tissue was undertaken on frozen sections (Vogelstein *et al.*, 1988). The diffuse nature of tumour infiltration in 27 cases meant that it was not possible to microdissect out small tumour foci in these samples, which were not used for further study. This left 43 tumours (numbered 1–43) and ten BPH (labelled A–J) controls for evaluation. Paired prostate and leucocyte samples were digested with proteinase K, then extracted with phenol, phenol/chloroform, chloroform and the DNA ethanol precipitated at −20°C (Sambrook *et al.*, 1989). Paired tumour/normal DNA samples were examined for loss of heterozygosity (LOH) at four loci within three introns (introns 1, 17 and 20) using two methods.

*LOH by restriction fragment length polymorphism (RFLP) analysis*

Restriction enzyme digest (*Rsa*I and *Bam*HI, Boehringer Mannheim) of 10 µg samples of DNA was followed by Southern blotting and hybridisation of the resulting filters (Hybond N, Amersham UK) with radiolabelled probes. Twenty-five ng of probe was labelled with [α-$^{32}$P]dCTP by the random primer techique (Feinberg & Vogelstein, 1984). Filters were hybridised overnight at 65°C in 0.23 M disodium hydrogen phosphate in 7% SDS. Sonicated human placental DNA was added as a blocking agent. They were then washed with solutions of increasing stringency and exposed to autoradiographic film. Two intragenic probes were used. Probe p68RS2.0 reveals a *Rsa*I polymorphism (eight alleles between 1.5 and 2.0 kb long) within a variable number tandem repeat (VNTR) sequence in intron 17 of the RB1 gene (Wiggs *et al.*, 1988). Probe p123m1.8 reveals a *Bam*HI polymorphism (two alleles, 4.5 and 2.2/2.3 kb long) to the 5′ end of the RB1 gene within intron 1 (Greger *et al.*, 1989).

*LOH using polymerase chain reaction (PCR)*

Primers to RB1 introns 17 (Greenwald *et al.*, 1992) and 20 (Onadim *et al.*, 1992) were used in PCR reactions to amplify across polymorphic regions. PCR to intron 17 reveals a *Xba*I polymorphism (two alleles, 190 and 123/55/12 bp long). PCR to intron 20 reveals a VNTR (26 alleles, from 300 to 350 bp long). Conditions were optimised for each set of primers used (Table I). By mixing DNA from homozygotes in different proportions, loss of heterozygosity with varying degrees of 'contamination' could be simulated, as described by McDaniel *et al.* (1991). Using this model we were able to check in preliminary studies that the PCR conditions used could detect LOH, even with significant benign tissue contamination.

A 100 ng sample of DNA was amplified, typical conditions being: reaction volume, 25 µl; *Taq* polymerase, 2 units (HT Technologies); primers, 60 ng each; and total NTPs, 0.4 mM. For intron 17, 23 cycles of amplification were used; for intron 20, 35 cycles. All PCR reactions were carried out in the presence of [α-$^{32}$P]dCTP, which was incorporated into the PCR products as described by Onadim *et al.* (1992). This technique allowed greater incorporation of radioactivity into the PCR products, enabling quicker visualisation of results. This method of labelling was also preferable to primer end labelling, as PCR followed by restriction enzyme digest would have given labelled restriction enzyme fragments of such disparate size that visual and densitometric analysis of results would have been difficult.

For intron 20, the PCR products were loaded directly into the wells. For intron 1 a 5 µl aliquot was digested overnight at 37°C with 20 units of *Xba*I and the resulting digest fragments loaded. Fragments were separated by electrophoresis in 6% non-denaturing polyacrylamide gels incorporating 10% glycerol (similar to those used for single-strand conformation polymorphism analysis). Gels were run at low power so that no heating occurred and the DNA strands did

**Table I** Details of polymerase chain reaction

| | |
|---|---|
| *RB intron 17* | 5′ Primer sequences 3′<br>CTGCAGTCCCACCTCAGCCTCCTTAGTAGA<br>GGATCCGCAGCTCTAGACTAATCCCAGCAC |
| Polymorphism | *Xba*I RFLP |
| Allele sizes | Two alleles, 190 and 123 + 55 + 12 base pairs |
| Cycles | 'Hot start', reaction heated to 95°C for 10 min before DNA added. Then:<br>1 × 95°C for 10 min, 62°C for 1 min, 72°C for 1 min<br>23 × 95°C for 1 min, 62°C for 1 min, 72°C for 1 min |
| *RB intron 20* | 5′ Primer sequences 3′<br>GTATGAACTCATGAGACAGGCAT<br>AATTAACAAGGTGTGGTGGTACACG |
| Polymorphism | VNTR |
| Allele sizes | 26 alleles, 300–350 base pairs long. |
| Cycles | 1 × 95°C for 15 min, 30 × 95°C for 20 s, 59°C for 20 s, 72°C for 60 s |

**Table II** Patients showing loss of heterozygosity for each locus examined

| | Locus | | | |
|---|---|---|---|---|
| *Patient no.* | *Intron 1 p123m1.8* | *Intron 17 PCR* | *Intron 17 p68RS2.0* | *Intron 20 PCR* |
| *Benign (BPH)* | | | | |
| A | – | – | – | ns |
| B | – | – | – | inf |
| C | – | np | inf | inf |
| D | – | np | inf | inf |
| E | DEL | DEL | inf | ns |
| F | inf | np | inf | ns |
| G | inf | np | inf | inf |
| H | – | np | inf | inf |
| I | inf | np | inf | inf |
| J | – | np | inf | ns |
| *Tumours* | | | | |
| T1M0 | | | | |
| 1 | – | – | DEL | DEL |
| T2M0 | | | | |
| 2 | – | inf | – | ns |
| 3 | – | inf | – | inf |
| 4 | inf | np | inf | ns |
| 5 | – | np | – | DEL |
| 6 | – | – | – | – |
| 7 | – | np | – | DEL |
| T2M1 | | | | |
| 8 | inf | np | inf | inf |
| 9 | – | np | – | ns |
| 10 | DEL | np | – | inf |
| 11 | – | – | DEL | DEL |
| 12 | inf | np | inf | DEL |
| T3M0 | | | | |
| 13 | – | inf | – | REAR |
| 14 | – | DEL | – | – |
| 15 | – | np | – | DEL |
| 16 | – | np | inf | inf |
| 17 | – | np | – | DEL |
| 18 | – | DEL | DEL | – |
| T3M1 | | | | |
| 19 | DEL | np | DEL | – |
| 20 | inf | np | inf | inf |
| 21 | inf | np | inf | – |
| 22 | inf | np | DEL | ns |
| 23 | – | np | inf | inf |
| 24 | – | DEL | – | ns |
| 25 | – | np | – | DEL |
| 26 | – | np | – | DEL |
| 27 | – | np | – | DEL |
| 28 | – | – | – | inf |
| 29 | inf | np | inf | inf |
| 30 | – | np | – | DEL |
| T4M0 | | | | |
| 31 | – | np | inf | – |
| 32 | – | – | – | ns |
| 33 | – | – | DEL | – |
| 34 | inf | DEL | DEL | ns |
| 35 | inf | np | inf | inf |
| T4M1 | | | | |
| 36 | DEL | DEL | DEL | ns |
| 37 | inf | np | inf | ns |
| 38 | – | np | inf | ns |
| 39 | inf | np | DEL | inf |
| 40 | DEL | DEL | DEL | ns |
| 41 | – | – | – | DEL |
| 42 | – | – | DEL | DEL |
| 43 | – | – | – | inf |

DEL, deletion; inf, informative, no LOH; –, non-informative; np, PCR amplification to RB intron 17 was not performed; ns, PCR reaction was not successful in amplifying both tumour and normal pair for RB intron 20; REAR, rearrangement with novel allele.

not separate. After electrophoresis the gels were dried and applied to autoradiographic film. Results were assessed visually and then checked using densitometry.

All tumour and BPH samples were hybridised with probes p123m1.8 and p68RS2.0 and amplified using PCR to intron 20. Patients who were non-informative to all of these three loci, were informative but showed no loss of heterozygosity, or were cases of special interest were additionally examined using PCR to intron 17. Cases showing deletions had the RFLP and/or PCR reactions repeated to confirm the result. Particular care was taken to confirm results in those patients in whom just one locus showed a deletion. All cases of LOH were confirmed using densitometry.

*Immunohistochemistry*

Nine patients with cancer and four with BPH were selected at random, and paraffin sections cut at 3 μm from their original histology blocks. The streptavidin–biotin complex method was used to demonstrate Rb protein. Sections were mounted on Vectabond (Vector laboratories) treated glass slides, dewaxed in xylene, then rehydrated through graded alcohols to water. The sections were then placed in a citrate buffer bath (pH 6) and microwaved (750 W) for 30 min (Norton, 1993). They were then rinsed with water, bathed in 1% hydrogen peroxide in methanol, rinsed in water and incubated with the mouse monoclonal antibody NCL-RB (NovoCastra Laboratories), diluted to optimum with 0.01 M PBS. After 1 h the sections were washed in 0.01 M PBS and the primary antiserum labelled with streptavidin–biotin complex–horseradish peroxidase (Duet Kit; K492, Dako). The horseradish peroxidase was visualised with 3,3′-diaminobenzidine tetrahydrochloride as a substrate and the nuclei counterstained for interpretation (Cattoretti *et al.*, 1992). Immunohistochemical staining showed pRB to be within the cell nucleus. In common with another immunohistochemical study of pRB using a different antibody (RB1-Ab20; Varley *et al.*, 1989), epithelial cells (tumour and benign), monocytes and endothelial cells may stain positively, but stromal cells do not. In those cells showing loss of nuclear pRB, some cytoplasmic staining was apparent. Benign epithelial cells staining positively within the nucleus sometimes also showed punctate staining within the cytoplasm. The sections stained for pRB by immunohistochemistry were assessed by the intensity of cell nuclear and cytoplasmic pRB staining and the percentage of cells showing loss of staining in these compartments for tumour, BPH, stroma and other benign tissue. Staining intensity was recorded as weak (+), moderate (+ +) and strong (+ + +). The percentage of cells with pRB nuclear or cytoplasmic staining was estimated as follows: loss in 95–100% (0), loss in 75–94% (1), loss in 50–74% (2) and loss in 0–49% (3).

### Results

*Patients and tumour stage and grade*

The age range of the 43 patients with prostate cancer was 55–88 (mean 72.8) years and of the ten with BPH was 61 to 80 (mean 71.9) years. The stages of the tumours were T1M0 ($n = 1$), T2M0 ($n = 6$), T2M1 ($n = 5$), T3M0 ($n = 6$), T3M1 ($n = 12$), T4M0 ($n = 5$ and T4M1 ($n = 8$). The tumours were graded as well ($n = 5$), moderately ($n = 14$) and poorly differentiated ($n = 24$).

*Loss of heterozygosity in cancer tissue*

Samples from 40 of 43 cancer patients were informative to one or more DNA probes/PCR sequences (Table II). Overall 24 (60%) out of 40 tumours showed loss of heterozygosity at one or more loci examined. Of these 24 patients, 4 (27%) out of 15 informative cases showed loss with probe p123m1.8, 11 (48%) out of 23 informative cases showed loss with probe p68RS2.0 and 13 (54%) out of 24 informative cases showed loss with PCR to RB1 intron 20 (Figure 1).

Twelve out of 24 cancers showing LOH exhibited a deletion at the only intron at which the case was informative.

**Figure 1** Examples of loss of heterozygosity. **a**, LOH in a prostate cancer using probe p123m1.8. **b**, LOH in cases of prostate cancer using probe p68RS2.0. **c**, LOH in cases of prostate cancer by PCR of intron 20. **d**, LOH in patient E with BPH only and also a case of prostate cancer by PCR of intron 17. N, normal DNA; T, tumour DNA; BPH, benign prostatic hypertrophy; bp, base pairs.

Seven cases showing LOH had losses at both of the two informative introns for that patient. The remaining five patients showed interstitial loss with preservation of part of the gene. Loss of the 5′ region with preservation of the 3′ end was shown by one tumour (number 10). Loss of the 3′ region with preservation of the 5′ end was found in three tumours. Loss of intron 17 with preservation of the flanking 5′ and 3′ ends was seen in one case (number 39). Examination of gel loading and comparison with the allele strength in informative cases gave no indication of any chromsomal duplications. One tumour (number 13) showed a rearrangement with novel allele formation on PCR to intron 20 (Figure 2). This PCR reaction was repeated with consistent results. All of the tumour/blood DNA samples have been examined at 22 different chromosomal loci (unpublished data), mostly VNTR polymorphisms. None of them, including tumour number 13, showed any disparity between the allele size displayed by tumour or blood DNA at these other loci, indicating that there had been no error in sample labelling during paired normal and tumour DNA extraction.

Prostate cancer is a diffusely infiltrating tumour, and the densitometry readings were used to assess the degree of background benign tissue contamination. Of those tumours showing LOH, 15 had a 70% or greater reduction in the signal strength of the deleted allele, six showed a 50–69% reduction and three showed a 30–40% reduction in the deleted allele. The last three cancers had a high precentage of benign tissue contamination despite tumour microdissection.

*Loss of heterozygosity in BPH tissue*

Of the nine informative benign (BPH) prostates, one showed LOH to intron 1 (*Bam*HI polymorphism) using probe p123m1.8 and intron 17 (*Xba*I polymorphism) as revealed by PCR (Figure 1d). This tissue showed retention of the 3′ locus within intron 17 as revealed by probe p68RS2.0. Intron 17 is large and the *Xba*I and VNTR polymorphism are 20 kb apart (Wiggs *et al.*, 1988). Densitometry showed a greater than 60% reduction in the size of the deleted allele from the BPH tissue when compared with its counterpart derived from blood.

Examination of the two loci showing loss in this patient was repeated five times with consistent results. All of the tissue resected from this 79-year-old patient (E) showed benign hyperplasia with both epithelial and stromal elements. There was no evidence of malignancy on histological examination, including the frozen section material used for DNA extraction. Despite the benign histology, this patient had a nodular prostate on rectal examination. A bone scan was performed, which was normal, and the prostate-specific antigen (PSA) was marginally elevated (20 ng $\mu l^{-1}$). The nodularity of the prostate and the elevated PSA strongly suggest an occult prostatic cancer. After reviewing the overall clinical picture in this patient, further biopsy of the prostate gland was not considered to be ethical, but he remains under careful clinical follow-up.

*Loss of heterozygosity* vs *stage and grade*

RB1 loss was similar between different stages and grades of tumour. LOH within tumours confined to the prostate (T1 and T2) was 60% (6/10); loss within those with extracapsular spread of tumour (T3 and T4) was also 60% (18/30). The M0 tumours showed LOH in 56% (9/16) cases; those with bone metastases (M1) showed loss in 62% (15/24) cases. Well-differentiated tumours showed loss in 80% (4/5) cases, moderately differentiated tumours showed loss in 71% (10/14) of cases and the poorly differentiated tumours showed LOH in 48% (10/21) of cases. There was no correlation between loss of RB1 and the grade or stage of tumour.

*Immunohistochemistry*

Of the nine tumour patients examined, four had shown a deletion of RB1, four were informative without LOH and one was non informative to all introns examined. Seven showed complete loss of pRB and two showed marked reduction of tumour nuclear staining (Table III). Seven of the tumours also contained areas of BPH within the sections examined, and a higher percentage and a greater intensity of nuclear staining was seen in the BPH cells than in adjacent tumour cell nuclei (Figure 3). All four cases with BPH alone showed greater staining than the tumours. There was no relationship between the proportion of nuclei showing loss of pRB staining and the reduction of the allele signal (as assessed by densitometry) in those showing LOH. This may have been because the material used for immunohistochemistry was from different prostatic curettings to those used for microdissection and DNA extraction.

### Discussion

Loss of heterozygosity within the RB1 gene was identified in 60% of prostate cancers, which is higher than has previously been reported (Carter *et al.*, 1990; Macoska *et al.*, 1992;

**Figure 2** Demonstration of a novel allele in tumour DNA by PCR of intron 20. N, normal DNA; T, tumour DNA; bp, base pairs.

**Table III** Immunohistochemistry results indicating percentage of cells showing loss of pRB staining

| *Patient no.* | *Tumour nucleus* | *Tumour cytoplasm* | *BPH nucleus* | *BPH cytoplasm* | *Stroma* |
|---|---|---|---|---|---|
| *Tumours* | | | | | |
| 8 | 1++ | 3++ | 3++ | 1+ | 0 |
| 9 | 0 | 3+++ | 3++ | 1+ | 0[a] |
| 18 | 0 | 0 | 1++ | 1+ | 0 |
| 21 | 1+++ | 2+ | NA | NA | 1+++[a] |
| 22 | 0 | 3++ | NA | NA | 0 |
| 31 | 0 | 3++ | 2++ | 1+ | 0 |
| 33 | 0 | 0 | 1++ | 0 | 0 |
| 35 | 0 | 0 | 1+++ | 0[b] | 0 |
| 42 | 0 | 2+ | 2+++ | 3+[b] | 0 |
| *BPH* | | | | | |
| A | NA | NA | 3+++ | 1+++[b] | 0 |
| C | NA | NA | 2+++ | 2+ | 0[a] |
| I | NA | NA | 2++ | 2+++[b] | 0 |
| J | NA | NA | 3+++ | 1+ | 0 |

[a]Endothelial, inflammatory and transitional epithelium cells with strong nuclear pRB staining. [b]Punctate pRB staining within cytoplasm. Percentage of cells showing loss of staining: 0, 95–100%; 1, 75–94%; 2, 50–74%; 3, 0–49%. Intensity of staining: +, weak; ++, moderate; +++, strong. NA, not applicable; BPH, benign prostatic hypertrophy.

**Figure 3** Demonstration of NCL-RB immunohistochemistry, showing an area of benign glands with retention of nuclear pRb (top) and an area of prostate tumour that has lost nuclear pRb expressions (bottom).

Sarkar *et al.*, 1992; Brooks *et al.*, 1993) and is similar to that for retinoblastoma tumours (Zhu *et al.*, 1992). Point mutations, not usually identified by RFLP studies, have been identified as the somatic mutation in retinoblastomas (Dunn *et al.*, 1988). In non-hereditary retinoblastoma, after excluding those with gross gene alterations by Southern blotting, point mutations of RB1 were found in all seven tumours examined (Yandell *et al.*, 1989).

Carter *et al.* (1990) reported losses of RB1 in 3 out of 13 prostate cancers when combining the results of the intragenic probe p68RS2.0 and a more distant probe to 13q. Results were not given independently for the intragenic and chromosomal arm probes. Brooks *et al.* (1993) showed 27% loss of RB1 in prostate cancer when examining RB1 intron 20 alone.

Sarkar *et al.* (1992) looked for deletions within the DNA of the RB1 promoter region and within exon 21 mRNA. They found an abnormal short-sized mRNA transcript of RB1 exon 21 extracted from a pure population of cells in tissue culture (cell line DU 145), but not in their seven cancer cases. No deletions within the promoter region were detected. The techniques used, however, would not have detected deletions lying outside the two loci studied or deletions of the whole of the promoter or exon 21 loci. If the whole of these loci had have been lost in the cases they studied, DNA or mRNA from benign tissue contamination would have been amplified to give a normal-sized band. Macoska *et al.* (1992) used only a single chromosome 13q marker and accepted a high degree of benign tissue contamination of tumour DNA. They found no losses in 19 informative cases, possibly because of benign DNA contamination and the fact that the probe used would not detect the small intragenic deletions that we and other workers have found within RB1.

The RB1 gene consists of 27 exons spread over 200 kb of genomic DNA (Hong *et al.*, 1989), which poses problems for the detection of small deletions. The cancer samples in this study were informative at one or more loci studied in 93% (40/43) of cases. Combining the results of all four loci gave a higher rate of allelic loss than the use of any single locus alone. There were 12 cases in which the only informative locus showed the allele loss. Small interstitial deletions of the RB1 gene have been widely reported in a number of different tumours, including bone and soft-tissue sarcomas (Wunder *et al.*, 1991), breast cancer (T'Ang *et al.*, 1988) and bladder cancer (Cairns *et al.*, 1991).

In seven of the nine tumours immunohistochemistry showed pRB loss in 95–99% of tumour nuclei. In the other two cases loss was seen in 75–94% of tumour nuclei. The presence of some pRB nuclear staining could be explained by the presence of mutant pRB which may be functionally inactive or by the retention a small clone of cells without pRB loss. This may suggest that loss of RB1, though early, is not the initiating event in carcinogenesis (Benedict *et al.*, 1990). Another possibility is that there is limited expression of the retained RB1 allele (possibly mutant), subject to activity of controlling genes.

One patient with BPH showed loss of heterozygosity of RB1, which may represent a premalignant field change occurring within the gland. de Vere White *et al.* (1992) noted abnormal RB1 mRNA expression in 1 of 13 benign prostates. Our case may be similar to this, though whether alteration of RB1 is representative of a premalignant change or has a role in prostatic adenoma or hyperplasia is unclear.

This study has shown that loss of RB1 gene is a frequent and possibly early event in prostatic tumorigenesis

We acknowledge the assistance given by Dr Paul Cairns and Mr John Gregory in this study. We thank the consultant urologists of the West Midlands Urological Research Group who kindly allowed us to study their patients. This work was funded by grants from the Former United Birmingham Hospitals Trustees, Zeneca Pharmaceuticals and The Imperial Cancer Research Fund.

**References**

BENEDICT, W.F., XU, H.-J., HU, S.-X. & TAKAHASHI, R. (1990). Role of the retinoblastoma gene in the initiation and progression of human cancer. *J. Clin. Invest.*, **85**, 988–993.

BLUTE, M.L., ZINCKE, H. & FARROW, G.M. (1986). Long term follow up of young patients with stage A adenocarcinoma of the prostate. *J. Urol.*, **136**, 840–843.

BOOKSTEIN, R., RIO, P., MADREPERLA, S.A., HONG, F., ALLRED, C., GRIZZLE, W.E. & LEE, W.H. (1990*a*). Promoter deletion and loss of retinoblastoma gene expression in human prostate cancer. *Proc. Natl Acad. Sci. USA*, **87**, 7762–7766.

BOOKSTEIN, R., SHEW, J.-Y., CHEN, P.-L. & SCULLY, P. (1990*b*). Suppression of tumourigenicity of human prostate carcinoma cell line by replacing a mutated RB gene. *Science*, **247**, 712–715.

BROOKS, J.D., BOVA, G.S., MARSHAL, F.F. & ISAACS, W.B. (1993). Allelic loss of the retinoblastoma gene in primary renal and prostate cancers. *J. Urol.*, **149**, 376A.

CAIRNS, P., PROCTER, A.J. & KNOWLES, M.A. (1991). Loss of heterozygosity at the RB locus is frequent and correlates with muscle invasion in bladder carcinoma. *Oncogene*, **6**, 2305–2309.

CARTER, B.S., EWING, C.M., WARD, W.S., TREIGER, B.F., AALDERS, T.W., SCHALKEN, J.A., EPSTEIN, J.I. & ISAACS, W.B. (1990). Allelic loss of chromosome 16q and 10q in human prostate cancer. *Proc. Natl Acad. Sci. USA*, **87**, 8751–8755.

CATTORETTI, G., BECKER, M.H.G., KEY, G., DUCHROW, M., SCHLUTER, C., GALLE, J. & GERDES, J. (1992). Monoclonal antibodies against recombinant parts of the Ki-67 antigen (MIB1 & MIB3) detect proliferating cells in microwave-processed formalin-fixed paraffin sections. *J. Pathol.*, **168**, 357-363.

DE VERE WHITE, R., ANDERSON, K.R., MEYERS, F.J., DEITCH, A.D., LEE, F., SIDERS, D.B., CHI, S.J. & GUMMERLOCK, P.H. (1992). Molecular abnormalities in benign prostatic hypertrophy. *J. Urol.*, **147**, 250A.

DUNN, J.M., PHILLIPS, R.A., BECKER, A.J. & GALLIE, B.L. (1988). Identification of germline and somatic mutations affecting the retinoblastoma gene. *Science*, **241**, 1797-1800.

FEINBERG, A.P. & VOGELSTEIN, B. (1984). A technique for radiolabelling DNA restriction endonuclease fragments of high specific activity. *Anal. Biochem.*, **137**, 266-267.

FRIEND, S.H., BERNARDS, R., ROGELJ, S., WEINBERG, R.A., RAPAPORT, J.M., ALBERT, D.M. & DRYJA, T.P. (1986). A human DNA segment with properties of the gene that predisposes to retinoblastoma and osteosarcoma. *Nature*, **323**, 643-646.

GEORGE, N.J.R. (1988). Natural history of localised prostate cancer managed by conservative therapy alone. *Lancet*, **i**, 494-497.

GREENWALD, B.D., HARPAZ, N., YIN, J., HUANG, Y., TONG, Y., BROWN, V.L., MCDANIEL, T.M., NAWKIRK, C., RESAU, J.H. & MELTZER, S.J. (1992). Loss of heterozygosity affecting the p53, Rb and mcc/apc tumour suppressor gene loci in dysplastic and cancerous ulcerative colitis. *Cancer Res..*, **52**, 741-745.

GREGER, V., PASSARGE, E., HOPPING, W., MESSMER, E. & HORSTHEMKE, B. (1989). Epigenetic changes may contribute to the formation and spontaneous regression of retinoblastoma. *Hum. Genet.*, **83**, 155-158.

HARBOUR, J.W., LAI, S.-L., WHANG-PENG, J., GAZDAR, A.F., MINNA, J.D. & KAYE, F.J. (1988). Abnormalities in structure and expression of the human retinoblastoma gene in SCLC. *Science*, **241**, 353-356.

HINDS, P.W., MITTNACHT, S., DULIC, V., ARNOLD, A., REED, S.I. & WEINBERG, R.A. (1992). Regulation of retinoblastoma protein functions by ectopic expression of human cyclins. *Cell*, **70**, 993-1006.

HOLUND, B. (1980). Latent prostate cancer in a consecutive autopsy series. *Scand. J. Urol. Nephrol.*, **14**, 29-35.

HONG, F.D., HUANG, H.J.S., TO, H., YOUNG, L.-J.S., ORA, A., BOOKSTEIN, R., LEE, E.Y.-H.P. & LEE, W.H. (1989). Structure of the human retinoblastoma gene. *Proc. Natl Acad. Sci. USA*, **86**, 5502-5506.

HUTCHINSON, G.B. (1981). Incidence and etiology of prostate cancer. *Urology*, **17**, 4-10.

LEE, E.Y.-H.P., TO, H., SHEW, J.-Y., BOOKSTEIN, R., SCULLY, P. & LEE, W.H. (1988). Inactivation of the retinoblastoma susceptibility gene in human breast cancers. *Science*, **241**, 218-221.

MCDANIEL, T.K., HUANG, Y., YIN, J., NEEDLEMAN, S.W. & MELTZER, S.J. (1991). Detection of loss of heterozygosity in tumor DNA samples by PCR. *Biotechniques*, **11**, 166-170.

MACOSKA, J.A., POWELL, I.J., SAKR, W. & LANE, M.-A. (1992). Loss of the 17p chromosomal region in a metastatic carcinoma of the prostate. *J. Urol.*, **147**, 1142-1146.

NORTON, A.J. (1993). Microwave oven heating for antigen unmasking in routinely processed tissue sections. *J. Pathol.*, **171**, 79-80.

ONADIM, Z., HUNGERFORD, J. & COWELL, J.K. (1992). Follow up of retinoblatoma patients having prenatal and perinatal predictions for mutant gene carrier status using intragenic polymorphic probes from the RB1 gene. *Br. J. Cancer*, **65**, 711-716.

PHILLIPS, S.M.A., MORTON, D.G., LEE, S.J., WALLACE, D.M.A. & NEOPTOLEMOS, J.P. (1994). Loss of heterozygosity of retinoblastoma and adenomatous polyposis susceptibility gene loci and in chromosomes 10p, 10q and 16q in human prostate cancer. *Br. J. Urol.*, **73**, 390-395.

SAMBROOK, J., FRITSCH, E.F. & MANIATIS, T. (1989). *Molecular Cloning. A Laboratory Manual.* Cold Spring Harbor Laboratory Press: Cold Spring Harbor, NY.

SARKAR, F.H., SAKR, W., LI, Y.W., MACOSKA, J. & CRISSMAN, J.D. (1992). Analysis of retinoblastoma (RB) gene deletion in human prostatic carcinoma. *Prostate*, **21**, 145-152.

T'ANG, A., VARLEY, J.M., CHAKRABORTY, S., MURPHREE, A.L. & FUNG, Y.-K.T. (1988). Structural rearrangement of the retinoblastoma gene in human breast cancer. *Science*, **242**, 263-266.

UICC (UNION INTERNATIONALE CONTRE LE CANCER) (1978). *TNM Classification of Malignant Tumours*, 3rd edn. International Union against Cancer: Geneva.

VARLEY, J.M., ARMOUR, J., SWALLOW, J.E., JEFFREYS, A.J., PONDER, B.A.J., T'ANG, A., FUNG, Y.-K.T., BRAMMAR, W.J. & WALKER, R.A. (1989). The retinoblastoma gene is frequently altered leading to loss of expression in primary breast tumours. *Oncogene*, **4**, 725-729.

VOGELSTEIN, B., FEARON, E.R., HAMILTON, S.R., KERN, S.E., PREISINGER, A.C., LEPPERT, M., NAKAMURA, Y., WHITE, R., SMITH, A.M.M. & BOS, J.L. (1988). Genetic alterations during colorectal-tumor development. *N. Eng. J. Med.*, **319**, 525-532.

VOGELSTEIN, B., FEARON, E.R., KERN, S.E., HAMILTON, S.R., PREISINGER, A.C., NAKAMURA, Y. & WHITE, R. (1989). Allelotype of colorectal carcinomas. *Science*, **244**, 207-211.

WIGGS, J., NORDENSKJOLD, M., YANDELL, D., RAPAPORT, J., GRONDIN, V., JANSON, M., WERELIUS, B., PETERSEN, R., CRAFT, A., RIEDEL, K., LIBERFARB, R., WALTON, D., WILSON, W. & DRYJA, T.P. (1988). Prediction of the risk of heterditary retinoblastoma, using DNA polymorphisms within the retinoblastoma gene. *N. Engl. J. Med.*, **318**, 151-157.

WUNDER, J.S., CZITROM, A.A., KANDEL, R. & ANDRULIS, I.L. (1991). Analysis of alterations in the retinoblastoma gene and tumour grade in bone and soft tissue sarcoma. *J. Natl Cancer. Inst..*, **83**, 194-200.

WYNDER, E.L., MABUCHI, K. & WHITMORE, W.F. (1971). Epidemiology of cancer of the prostate. *Cancer*, **28**, 344-360.

YANDELL, D.W., CAMPBELL, T.A., DAYTON, S.H., PETERSEN, R., WALTON, D., LITTLE, J.B., MCCONKIE-ROSELL, A., BUCKLEY, E.G. & DRYJA, T.P. (1989). Oncogenic point mutations in the human retinoblastoma gene: their application to genetic counselling. *N. Engl. J. Med.*, **321**, 1698-1695.

YUNIS, J.J. & RAMSAY, N. (1978). Retinoblastoma and subband deletion of chromosome 13. *Am. J. Dis. Child.*, **132**, 161-163.

ZARIDZE, D.G. & BOYLE, P. (1987). Cancer of the prostate: epidemiology and aetiology. *Br. J. Urol.*, **59**, 493-502.

ZHU, X., DUNN, J.M., GODDARD, A.D., SQUIRE, J.A., BECKER, A., PHILLIPS, R.A. & GALLIE, B.L. (1992). Mechanisms of loss of heterozygosity in retinoblastoma. *Cytogenet. Cell. Genet.*, **59**, 248-252.